# Copper(II) complexes with derivatives of pyrazole as potential antioxidant enzyme mimics

**DOI:** 10.1007/s00044-012-0233-5

**Published:** 2012-09-26

**Authors:** Bogumiła Kupcewicz, Krzysztof Sobiesiak, Katarzyna Malinowska, Kamila Koprowska, Malgorzata Czyz, Bernhard Keppler, Elżbieta Budzisz

**Affiliations:** 1 Department of Inorganic and Analytical Chemistry, Faculty of Pharmacy, Collegium Medicum in Bydgoszcz, Nicolaus Copernicus University in Torun, Curie-Sklodowskiej 9, 85-094 Bydgoszcz, Poland; 2 Department of Chemistry and Clinical Biochemistry, Medical University of Lodz, Haller Square 1, 90-647 Lodz, Poland; 3 Department of Molecular Biology of Cancer, Medical University of Lodz, Mazowiecka 6/8, 92-215 Lodz, Poland; 4 Institute of Inorganic Chemistry, University of Vienna, Währingerstr. 42, 1090 Vienna, Austria; 5 Department of Cosmetic Raw Materials Chemistry, Faculty of Pharmacy, Medical University of Lodz, Muszynskiego 1, 90-151 Lodz, Poland

**Keywords:** Enzyme mimic, Copper(II) complexes, Pyrazoles, Reactive oxygen species, Cyclic voltammetry

## Abstract

A series of six mononuclear Cu(II) complexes with pyrazole-based ligands: 5-(2-hydroxybenzoyl)-3-methyl-1-(2-pyridinyl)-1*H*-pyrazol-4-phosphonic acid dimethyl ester (**1a**), 5-(2-hydroxyphenyl)-3-methyl-1-(2-pyridylo)-1*H*-pyrazole-4-carboxylic acid methyl ester (**1b**) and 1-benzothiazol-2-yl-5-(2-hydroxyphenyl)-3-methyl-1*H*-pyrazole-4-carboxylic acid methyl ester (**1c**) were characterized regarding to electrochemical and antioxidant properties. All complexes exhibit suitable Cu(II)/Cu(I) redox potential (*E*_1/2_) to act as antioxidant enzymes mimic. The five of these complexes were found to be trifunctional enzyme mimics possessing SOD, CAT and GPx-like catalytic activities. Moreover, Cu(II) complexes were capable to decrease ROS level in melanoma cells and observed effects were not merely a reflection of cytotoxicity.

## Introduction

Reactive oxygen species (ROS) such as O_2_^−^, H_2_O_2_ and ^•^OH are generated in cells through aerobic metabolic processes or as a result of interaction with exogenous agents. Low levels are essential for proper cell function, but excess levels of ROS are responsible for ‘oxidative stress’ which has been linked with the progression of ageing and many human diseases, e.g. neurogenerative, cardiovascular and cancer. Superoxide dismutases (SODs), catalase (CAT) and glutathione peroxidase (GPx) are enzymes which act as a primary cellular defence system against oxidative damage in living organisms.

Copper(II) has an important biological role in all living systems as an essential trace element (Linder and Hazegh-Azam, 1996). The Cu(II) complexes with organic ligands have been used as analgesic, antipyretic, antiinflammatory and a platelet anti-aggregating agents. Due to the redox behaviour of the Cu(II)/Cu(I) system and the interaction of copper complexes with O_2_ biomimetic complexes of copper ions with biologically interesting ligand have been investigated in detail. They have antioxidant, antitumor activity and protect against some injuries being consequences of UV exposure (Zheng *et al*., 2006). Recently, several reports have appeared in the literature describing the anticancer activity of Cu(II) derivatives of many classes of nitrogen donors including thiosemicarbazone, imidazole (Huang *et al*., 2005). Among them, pyrazole-containing complexes have been reported to possess antitumor activity which is comparable to that of cisplatin (Sakai *et al*., 2000; Wheate *et al*., 2001; Al-Allaf and Rashan, 2001). In addition, considerable interest in the pyrazole moiety has been stimulated by promising pharmacological, agrochemical and analytical applications of pyrazole-containing derivatives (Eicher and Hauptmann, 1995; Eliguero *et al*., 1997; Onoa *et al*., 1999, 2002; Duivenvoorden *et al*., 2005). Recently, substituted pyrazoles have been used as analytical reagents in the complexation of transition metal ions (Wisniewski *et al*., 1994; Majsterek *et al*., 2011).

In our previous articles, we have investigated the synthesis, X-ray structures, physicochemical properties and preliminary cytotoxic effect for Cu(II) complexes with pyrazole derivatives as ligands (Miernicka *et al*., 2008; Budzisz *et al*., 2009, 2010).

Here, we present evaluation of the antioxidant activity of six Cu(II) complexes with three ligands: 5-substituted-3-methyl/phenyl-1-(2-pyridinyl)-1*H*-pyrazol-4-carboxylic acid methyl ester (**1a**) or phosphonic acid dimethyl ester (**1b**) and 1-benzothiazol-2-yl-5-(2-hydroxyphenyl)-3-methyl-1*H*-pyrazole-4-carboxylic acid methyl ester (**1c**). We assessed the ability to act these complexes as SOD, CAT and GPx enzyme mimics and to scavenge ROS.

## Experimental

### Materials and methods

The ligands and complexes with Cu(II) ions were prepared as described elsewhere (Miernicka *et al*., 2008; Budzisz *et al*., 2009, 2010). All substances were reagent grade or better and were used without further purification.

### Trolox equivalent antioxidant capacity (TEAC) assay with ABTS and K_2_ S_2_O_8_

The main mechanism of this test is the reduction of the ABTS (2,2′-azino-bis[3-ethylbenzothiazoline-6-sulphonate]) radical cation by antioxidants. The ABTS radical cation was obtained as a result of reaction of ABTS stock solution (7 mM in water) with 2.45 mM potassium persulfate. For measurements, the ABTS^•+^ solution was diluted with ethanol to an absorbance of 0.700 ± 0.020 at 754 nm. Stock solutions of the all compounds were diluted with DMSO. For the photometric assay 1,350 μL of the ABTS^•+^ solution and 150 μL of antioxidant solution were mixed for 45 s and absorbance was measured immediately after 1 min at 754 nm. The concentration of Cu(II) complexes was varied in the range 2–400 μM. The antioxidant activity of the tested compounds was calculated by determining the decrease in absorbance at different concentrations by using the following equation (Schlesier *et al*., 2002): %antioxidant activity = ((*E*_ABTS_^•+^ − *E*_Standard_)/*E*_ABTS_^•+^) × 100.

### Blood sample preparation and enzymes activity measurement

Examinated group comprise 50 individuals (aged 27–45 years). Blood was taken from cubital vain on heparinized sample (5 mL). Blood was centrifuged 10 min at 3,000 rpm in room temperature. Obtained erythrocytes were three times washed 0.9 % sol NaCl at the same condition of centrifugation. After centrifugation and removal of the supernatant 920 μL of sample and 80 μL of Cu(II) complex solution were mixed. Next it was added to 1 mL glucose and incubated at 37 °C, after which the hemolysate were prepared and then frozen at −70 °C. Thus, prepared hemolysate was used for further experiments. The concentration of compounds **2a**–**c** and **3a**–**c** in experiment was 25 μg/mL of blood.

Activity of CAT, GPx, SOD enzymes and TAS value were determined in blood samples (erythrocytes) treated by Cu(II) complexes and in control samples using spectrophotometric methods. All absorbance measurements were performed with a UV/Vis Spectrometer Lambda 14P (Perkin Elmer, USA).

CAT activity in erythrocytes was determined according to spectrophotometric procedure by Beers and Sizer (1952) and expressed in Bergmeyer units (BU/g Hb). CAT activity was measured at 25 °C by recording H_2_O_2_ decomposition at 240 nm. One BU of CAT activity is defined as the amount of enzyme decomposing 1 g of H_2_O_2_/min.

GPx activity in erythrocytes was measured according to Little and O’Brien (1968) methods and expressed in enzymatic units (U/g Hb). The difference in the rate of GPx reaction with glutathione and lumen in the sample is used for its activity determination by absorbance measurement at 412 nm. One unit of GPx activity is calculated as an amount of enzyme which causes 10 % decrease of the level of reduced glutathione within 1 min at 25 °C, pH 7.0.

SOD activity in erythrocytes was measured according to Misra and Fridovich (1972) methods. The activity was determined at 37 °C by the absorbance increase at 480 nm. Activity of SOD was expressed in adrenaline units (U/g Hb/100 mL). Haemoglobin concentrations were carried out according to Van Kempen and Zijlstra (1961).

### Total antioxidant status determination

Determination of the total antioxidant status in blood plasma was performed by spectrophotometric method according to procedure no. NX2332 by Randox (Randox Laboratories Ltd., United Kingdom,). In brief, ABTS (2,2′-azino-di-[3-ethylbenzthiazoline sulphonate]) was incubated with peroxide (metmyoglobin) and H_2_O_2_ to produce the radical cation ABTS with a relatively stable blue-green colour. Antioxidants when added in examined sample caused suppression of this colour production measured as decrease of absorbance with a spectrometer (UV/Vis Spectrometer Lambda 14P, Perkin Elmer, USA) at 600 nm. The total antioxidant status was calculated as concentration of antioxidants (mM).

### The electrochemical properties

The electrochemical properties of ligands and metal ion complexes have been studied by cyclic voltammetry in DMF solution. Voltammetric measurements were made with the aid PGSTAT12 AUTOLAB electrochemical analyzer. Three electrodes were utilized in this system, a glassy carbon working electrode (GCE), a platinum wire auxiliary electrode and silver wire in contact with 0.1 M AgNO_3_ in ACN reference electrode. The GCE with 3.0-mm diameter was manually cleaned with 1 µm alumina polish prior each scan. All solutions were deareated for 10 min prior to measurements with pure argon and then a blanket atmosphere of argon was maintained over the solution during measurements. The potentials were measured in 0.2 M [*n*Bu_4_N][BF_4_]/DMF as supporting electrolyte, using the [Fe(*η*5-(C_5_H_5_)_2_] in DMF (*E*_1/2_ = +0.72 V) as internal standard.

### Cell viability

Cell viability was determined after 44 h of culturing of A375 cells in the presence of tested compounds at indicated concentrations. An acid phosphatase activity (APA) assay was used to assess viable cell numbers in cultures. In brief, the plates were centrifuged at the indicated time points, the medium was discarded and replaced with 100 μL assay buffer containing 0.1 M sodium acetate (pH 5), 0.1 % Triton X-100 and 5 mM *p*-nitrophenyl phosphate (pNPP; Sigma-Aldrich, St. Louis, MO) and incubated for additional 2 h at 37 °C. The reaction was stopped with 10 μL of 1 M NaOH, and the absorbance values were measured at the wavelength of 405 nm using a microplate reader (Infinite M200Pro, Tecan, Austria).

### Measurement of intracellular ROS

ROS levels were evaluated by flow cytometry using the probe 2′,7′-dichlorodihydrofluorescein diacetate (H_2_DCF-DA; Sigma-Aldrich, St. Louis, MO, USA) as described previously (Lesiak *et al*., 2010). In brief, A375 melanoma cells (a gift from Prof. Piotr Laidler, Jagiellonian University, Poland) were seeded into a 12-well plate and cultured for 18 h in RPMI-1640 medium with 5 % foetal bovine serum. On the day of experiment, the cells were treated with complexes at indicated concentrations for 1 h. An equivalent concentration of DMSO was used in the control culture. In all experiments, incubation with 2 mM *N*-acetylcysteine (NAC) for 1 h was used as a reference control. After treatment, cells were collected, washed with PBS and incubated with the 5 μM H_2_DCF-DA at 37 °C for 30 min in the dark. Immediately after staining cells were collected and analyzed by flow cytometry (FACSCalibur; Becton–Dickinson, Mountain View, CA, USA). All results were processed by using CellQuest software (Becton–Dickinson).

## Results and discussion

### Chemistry

**Fig. 1:**
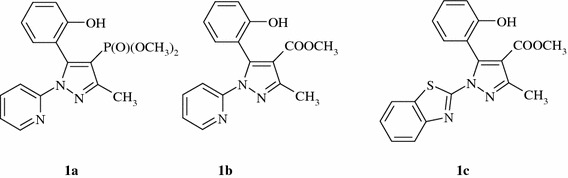
Structure of the ligands

**Fig. 2:**
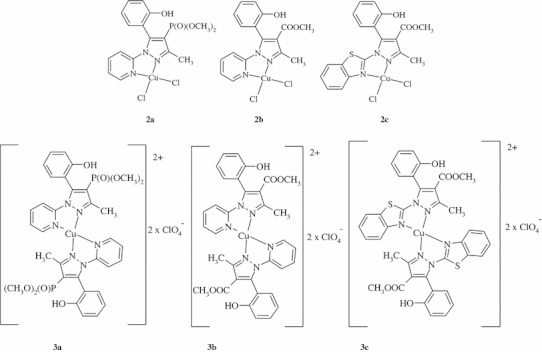
Proposed structures of the **2a**–**c** and **3a**–**c** complexes

We have prepared a two series of Cu(II) complexes with a substituted pyrazoles (**1a**–**c**), as depicted in Fig. 1. Complexes **2a**–**c** of the general formula (CuLCl_2_) were obtained in reaction of ligands with CuCl_2_·2H_2_O (in a 1:1 molar ratio) in ethyl acetate. The complexes **3a**–**c** were synthesized in molar ratio 2:1 giving ionic complexes of general formula [CuL_2_](ClO_4_)_2_ (Fig. 2). The details of synthesis, results of elemental analysis and characterization of complexes using IR, NMR and MS spectroscopy were described in our previous articles (Miernicka *et al*., 2008; Budzisz *et al*., 2009, 2010). All complexes were recrystallized from DMF, but only compounds **2a**–**c** yielded crystals suitable for X-ray diffraction. The complexes exhibit trigonal bipyramidal configuration at Cu(II) centre.

### SOD/CAT/GPx-like activity

**Fig. 3:**
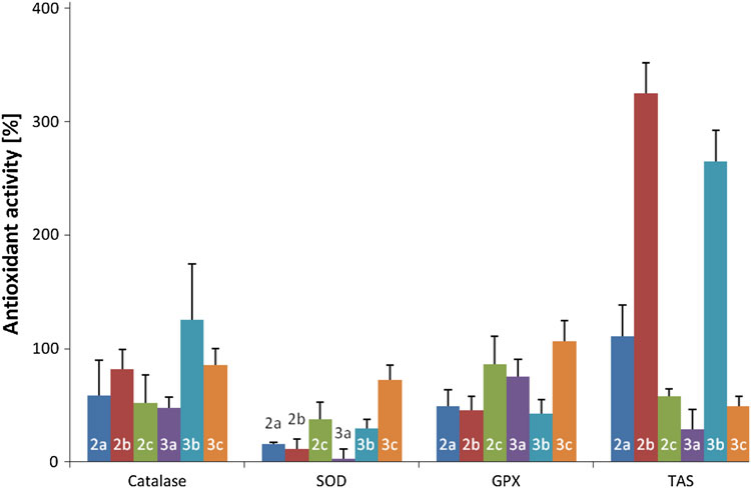
The enhancement (in %, mean value + SEM) of antioxidant activity of catalase, superoxide dismutase (SOD), glutathione peroxidase (GPx) and total antioxidant status (TAS) value in blood samples treated with Cu(II) complexes (20 μg/mL of blood) in comparison with antioxidant activity in control samples

Complexes **2a**–**c** and **3a**–**c** were investigated on their antioxidant activity. The SOD (SOD-1), GPx and CAT activities and moreover total antioxidative status (TAS) have been determined. The results were expressed as enhancement (in %) of antioxidant enzymes activity and TAS value in blood samples treated with Cu(II) complexes in comparison to antioxidant activity in control samples and are presented in Fig. 3.

Our data underline that addition of compounds in blood lead to statistically significant increase in enzymes activities in comparison to control samples. The differences between two groups (samples with synthesized compounds and control group) were calculated using *t* test for dependent samples. *T* test results indicate that activity of CAT, SOD, GPx and TAS value in all samples with metal complexes was statistically significant (*p* < 0.01) greater than in control samples.

SOD-1 is metalloprotein that catalyze ‘dismutation’ reaction which detoxify superoxide radicals (O_2_^•−^) (Ercal *et al*., 2001):

$$ [2{\text{O}}_2^{ \bullet - } + 2{{\text{H}}^+ } \to {{\text{H}}_2}{{\text{O}}_2} + {{\text{O}}_2}.] $$

The mechanism proposed for the dismutation of superoxide anions by both SOD and metal complexes is thought to involve redox reactions with Cu(II) and Cu(I) ions (Ercal *et al*., 2001; Patel *et al*., 2009):

$$ [{\text{C}}{{\text{u}}^{2 + }} + {\text{O}}_2^{ \bullet - } \to {\text{C}}{{\text{u}}^+ } + {{\text{O}}_2}] $$

$$ [{\text{C}}{{\text{u}}^+ } + {\text{O}}_2^{ \bullet - } + 2{{\text{H}}^+ } \to {\text{C}}{{\text{u}}^{2 + }} + {{\text{H}}_2}{{\text{O}}_2}.] $$

The addition of Cu(II) complexes to blood samples result in statistically significant increase of SOD activity (*p* < 0.01) in case of all compounds. The level of SOD was increased in order **a** < **b** < **c** in both series of complexes, 16.00 < 28.00 < 38.42 % and 3.85 < 33.03 < 59.16 % for series **2** and **3**, respectively. The comparison of complexes with the same ligands revealed statistically significant difference only between **2a** and **3a** complexes (*p* < 0.001).

CAT and GPx are enzymes which disproportionate H_2_O_2_ by converting it into the H_2_O and O_2_ (CAT) or only into the water (GPx) (Day, 2009).

$$ [{{\text{H}}_2}{{\text{O}}_2} \to {{\text{O}}_2} + {{\text{H}}_2}{\text{O}}] $$

$$ [2{\text{GSH}} + {{\text{H}}_2}{{\text{O}}_2} \to {\text{GS-SG}} + 2{{\text{H}}_2}{\text{O}} .] $$

In the present findings, all six Cu(II) complexes induced a significant (*p* < 0.01) increase (from 45 to 126 % more than in control samples) in antioxidant enzymes levels of GPx and CAT.

When SOD activity is high, the conversion of superoxide anion (O2^•−^) to hydrogen peroxide (H_2_O_2_) is facilitated. High SOD activity in conjunction with low GPx activity will lead to increased levels of H_2_O_2_ and H_2_O_2_-derived reactive species such as hydroxyl radical (^•^OH). Relationship between SOD and CAT + GPx can affect more on cell sensitivity to a free radical attack than absolute amounts of the individual antioxidant enzymes. Low ratio of SOD/CAT + GPx demonstrates high cell resistance to oxidative damage.

The ratio between SOD activity and the activities of CAT + GPx that remove the H_2_O_2_ formed by SOD was from 6.06 to 37.55 % lower in samples treated by Cu(II) complexes than in control samples. These results indicated that all complexes are more efficient in reduction of H_2_O_2_ than scavenging of superoxide radicals. In the series **3** of complexes SOD/(CAT + GPx) ratio decreased in order: **a** > **b** > **c** and is very good correlated with Cu(II)/Cu(I) redox potential.

### Free radical and ROS scavenging ability of the complexes

The antioxidant activity of Cu(II) complexes can also be expressed as TEAC, which means the concentration (mM) of Trolox whose antioxidant activity are identical to 1 mg of the complexes themselves. Trolox used as a standard is a derivative of vitamin E, strong natural antioxidant. The TEAC value reveal the relative ability of hydrogen- or electron-donating antioxidants to scavenge the ABTS^•+^ radical cation compared with that of Trolox. The results obtained for complexes with Cu(II) ions are summarized in Table 1.

**Table 1:** Antioxidant activity of complexes based on ABTS^•+^ assay (absorbance was measured at 734 nm, 5 min after initial mixing)

Compounds	IC_50_ (mM)	TEAC (mM)
**2a**	5.88 ± 0.59	0.12
**2b**	0.11 ± 0.00	0.27
**2c**	1.56 ± 0.12	0.14
**3a**	9.62 ± 2.13	0.11
**3b**	>100	<0.06
**3c**	10.04 ± 0.26	0.13
Trolox	0.136 ± 0.05	

Data expressed as mean value ± SD of triplicate measurements

*TEAC* Trolox equivalent antioxidant capacity, expressed as mmol Trolox/mg of complex

ROS levels were also evaluated by flow cytometry using the probe H_2_DCF-DA. This non-polar compound diffuses into cells, where undergoes deacetylation by cytosolic esterases to form the non-fluorescent polar derivative DCFH and thereby is trapped within the cells. In the presence of intracellular H_2_O_2_, DCFH is oxidized to the highly fluorescent DCF. Cells were untreated or exposed to selected concentrations (1 or 20 μM) of Cu(II) complexes for 1 h and then stained with 5 μM H_2_DCF-DA for 30 min. The test was carried out in duplicate.

**Fig. 4:**
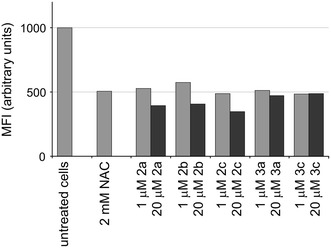
Effects of Cu(II) complexes on intracellular ROS level in A375 melanoma cells

**Fig. 5:**
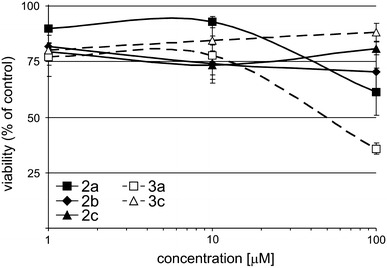
Cu(II) complexes decreased the number of viable cells in melanoma cultures. An APA assay was used to assess changes in viable cell numbers. Melanoma cell line A375 was cultured with complexes at the indicated concentrations for 44 h. Viable cell numbers in drug-treated cultures were expressed as the percentages of cell number in the control culture. Data represent the mean ± SD of three measurements

When A375, a highly aggressive melanoma cell line were treated with Cu(II) complexes, a marked reduction of H_2_O_2_ levels was observed, irrespective of the structure of tested compounds. Measurements of fluorescence revealed that Cu(II) complexes reduced intracellular H_2_O_2_ in melanoma cells to the level similar as obtained in the presence of NAC, well known for its high antioxidant activity. NAC (2 mM) which was used as a reference control induced 50 % decline in fluorescence intensity in comparison to untreated cells, whereas Cu(II) complexes at 20 μM caused 40–49.5 % decrease in fluorescence intensity (Fig. 4). At that concentration Cu(II) complexes were not highly toxic to melanoma cells as they reduced the viable cell number to 70–85 % of that observed in control culture even when incubation was prolonged to 44 h (Fig. 5). Thus, the observed effects were not mainly due to cytotoxicity of Cu(II) complexes.

**Fig. 6:**
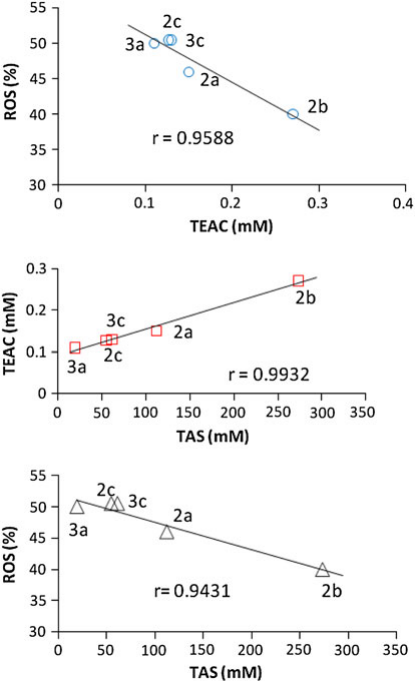
TAS–TEAC, TAS–ROS and TEAC–ROS relationships

The ROS-scavenging potential, TAS and TEAC values of five Cu(II) complexes were compared each other and the very good linear correlation were obtained (**3b** complex was excluded due to inconsistent results of Trolox assay). Correlation coefficient (*r*) values were: 0.9932, 0.9431 and 0.9588 for TAS–TEAC, TAS–ROS and TEAC–ROS relationships, respectively (Fig. 6).

### Cyclic voltammetry

Electrochemical properties of the complex series were investigated with cyclic voltammetry in DMF solution. The results obtained in the electrochemical studies are summarized in Table 2. The complexes were also studied under the same conditions for a direct comparison of the results. All complexes show one-electron redox wave in the plotted potential range, attributed to the Cu(II)/Cu(I) redox couple. Second pair of peaks was only observed in the case of **1c** compound. For four of them (**1a**, **1b**, **2b** and **3b**) only single reduction waves were present additionally. The *E*_1/2_ values are within the range of −0.538 V (**1b**) to 0.076 V (**2c**). A considerable dispersion of *E* values was observed. It is possible to observe that *E* values are increasing in the following row: **a** < **b** < **c** for ligands and **2** series of complexes. However, for **3** series of complexes there is an inverse relationship: **c** < **b** < **a**. In case of complexes with **1a** ligand (**2a** and **3a**), one observes peak separation of roughly 45 mV, in contrast to complexes with ligands **1b** and **1c** which exhibit three times greater peak separation (130–190 mV). The peak-to-peak separation (Δ*E*_p_) and proportion of the anodic peak current and the cathodic peak current mostly indicates a quasireversible process. However, in the case of **1a**, **2a** and **3a** compounds, there is a reversible process.

**Table 2:** Cyclic voltammetry data (V)

No of compounds	*E*_pa_^1^	*E*_pc_^1^	*E*_1/2_^1^	*E*_pa_^2^	*E*_pc_^2^	*E*_1/2_^2^
**1a**	0.081	−0.344	−0.131	–	–	–
**1b**	−0.400	−0.675	−0.538	−0.287^a^	–	–
**1c**	0.097	−0.014	0.042	−0.034	−0.380	−0.207
**2a**	−0.216	−0.264	−0.250	–	–	–
**2b**	−0.219	−0.349	−0.284	0.043^a^	–	–
**2c**	0.158	−0.005	0.076	–	–	–
**3a**	0.123	−0.082	0.021	–	–	–
**3b**	−0.148	−0.339	−0.244	0.225^a^	–	–
**3c**	−0.229	−0.400	−0.315	–	–	–

^a^Only anodic peak

It is known that an adequate Cu(II)/Cu(I) redox potential for effective catalysis of superoxide radical must be required between −0.405 V for O_2_/O_2_^•−^ and +0.645 V for O_2_^•−^/H_2_O_2_ versus SCE (at pH 7) or between −0.762 and +0.29 V versus Ag/AgNO_3_/ACN, respectively. The Cu(II)/Cu(I) redox couples of both series of complexes (**2a**–**c**, **3a**–**c**) are within this potential range; therefore, these complexes are expected to exhibit SOD-like activity. The highest enhancement of SOD activity exhibits complexes with ligand **1c** (**2c**, **3c**).

To make a Cu(II) complex thermodynamically competent in the H_2_O_2_ detoxification, the redox potential of the metal-centred redox couples should fall within the 0.04 V (O_2_/H_2_O_2_) to 1.01 V (H_2_O/H_2_O_2_) versus SCE potential range or between −0.32 and 0.65 V versus Ag/AgNO_3_ electrode. All the complexes (**2a**–**c**, **3a**–**c**) have suitable *E*_*1*/2_ potential and showed activity for the catalytic decomposition of H_2_O_2_. Among them **2a**, **2b**, **3b** and **3c** complexes are comparably effective as CAT mimics.

## Conclusions

In this study, electrochemical and antioxidant properties of six Cu(II) mononuclear complexes with pyrazole-based ligands were evaluated. The majority of Cu(II) complexes, under the experimental conditions used in this study, were found to be trifunctional enzyme mimics possessing SOD, CAT and GPx-like catalytic activities. They may react with superoxide as well as with product of superoxide dismutation, H_2_O_2_. The only **3a** complex showed negligible SOD-like activity but moderate ability to reduction H_2_O_2_.

Moreover, Cu(II) complexes were capable to decrease ROS level in melanoma cells. Those cells constantly exposed to oxidative stress induced by UV radiation and quinone toxicity from melanin synthesis are very efficient in scavenging ROS. Thus, the capacity of tested compounds to neutralize hydrogen peroxide was shown to substantially support natural mechanisms existing in those cells.

## References

[CR1] Al-Allaf TAK, Rashan LJ (2001). Stereochemistry—cis- and trans-platinum and palladium complexes: a comparative study review as antitumour agents. Boll Chim Farm.

[CR2] Beers R, Sizer T (1952). A spectrophotometric method for measuring the breakdown of hydrogen peroxide by catalase. J Biol Chem.

[CR3] Budzisz E, Miernicka M, Lorenz IP, Mayer P, Krajewska U, Rozalski M (2009). Synthesis and X-ray structure of platinum(II), palladium(II) and copper(II) complexes with pyridine–pyrazole ligands: influence of ligands structure on cytotoxic activity. Polyhedron.

[CR4] Budzisz E, Miernicka M, Lorenz IP, Mayer P, Balcerczak E, Krajewka U, Rozalski M (2010). Synthesis, X-ray structures and cytotoxic activity of platinum(II), palladium(II) and copper(II) complexes with chelating ligands. Eur J Med Chem.

[CR5] Day BJ (2009). Catalase and glutathione peroxidase mimics. Biochem Pharmacol.

[CR6] Duivenvoorden WCM, Liu Y, Schatte G, Kraatz HB (2005). Synthesis of redox-active ferrocene pyrazole conjugates and their cytotoxicity in human mammary adenocarcinoma MCF-7 cells. Inorg Chim Acta.

[CR7] Eicher T, Hauptmann S (ed) (1995) The chemistry of heterocycles structure, reaction synthesis and applications (trans: H. Suschitzky, J. Suschitzky) Georg Thime Verlag, Stuttgart, p 184

[CR8] Eliguero J, Katritzky AR, Pees CW, Scriven EF (1997). Comprehensive heterocyclic chemistry II.

[CR9] Ercal N, Gurer-Orhan H, Aykin-Burns N (2001). Toxic metals and oxidative stress part I: mechanisms involved in metal induced oxidative damage. Curr Top Med Chem.

[CR10] Huang R, Wallqvist A, Covell DG (2005). Anticancer metal compounds in NCI’s tumor-screening database: putative mode of action. Biochem Pharmacol.

[CR11] Lesiak K, Koprowska K, Zalesna I, Nejc D, Düchler M, Czyz M (2010). Parthenolide, a sesquiterpene lactone from the medical herb feverfew, show anticancer activity against human melanoma cells in vitro. Melanoma Res.

[CR12] Linder MC, Hazegh-Azam M (1996). Copper biochemistry and molecular biology. Am J Clin Nutr.

[CR13] Little C, O’Brien P (1968). An intracellular GSH peroxidase with a lipid peroxide substrate. Biochem Biophys Res Commun.

[CR14] Majsterek I, Malinowska K, Stanczyk M, Kowalski M, Blaszczyk J, Kurowska AK, Kaminska A, Szaflik J, Szaflik JP (2011). Evaluation of oxidative stress markers in pathogenesis of primary open-angle glaucoma. Exp Mol Pathol.

[CR15] Miernicka M, Szulawska A, Czyz M, Lorenz IP, Mayer P, Karwowski B, Budzisz E (2008). Cytotoxic effect, differentiation, inhibition of growth and crystal structure of *N*,*N*-donor ligand and its palladium(II), platinum(II) and copper(II). J Inorg Biochem.

[CR16] Misra HP, Fridovich J (1972). The role of superoxide anion in the autooxidation of epinephrine and a simple assay superoxide dismutase. J Biol Chem.

[CR18] Onoa GB, Moreno V (2002). Study of the modifications caused by cisplatin, transplatin, and Pd(II) and Pt(II) mepirizole derivatives on pBR322 DNA by atomic force microscopy. Int J Pharm.

[CR17] Onoa GB, Moreno V, Font-Bardia M, Solans X, Perez JM, Alonso C (1999). Structural and cytotoxic study of new Pt(II) and Pd(II) complexes with the bi-heterocyclic ligand mepirizole. J Inorg Biochem.

[CR19] Patel RN, Shukla KK, Singh A, Choudhary M, Chauhan UK, Dwivedi S (2009). Copper(II) complexes as superoxide dismutase mimics: synthesis, characterization, crystal structure and bioactivity of copper(II) complexes. Inorg Chim Acta.

[CR20] Sakai K, Tomista Y, Ue T, Goshima K, Ohminato M, Tsubomura T, Matsumoto K, Ohmura K, Kawakami K (2000). Syntheses, antitumor activity, and molecular mechanics studies of *cis*-PtCl_2_(pzH)_2_ (pzH = pyrazole) and related complexes. Crystal structure of a novel Magnus-type double-salt [Pt(pzH)_4_][PtCl_4_][*cis*-PtCl_2_(pzH)_2_]_2_ involving two perpendicularly aligned 1D chains. Inorg Chim Acta.

[CR21] Schlesier K, Harwat M, Böhm V, Bitsch R (2002). Assessment of antioxidant activity by using different in vitro methods. Free Radic Res.

[CR22] Van Kempen EJ, Zijlstra WG (1961). Standarization of hemoglobinometry II. The hemoglobincyanide method. Clin Chim Acta.

[CR23] Wheate NJ, Cullinane C, Webster LK, Collins JG (2001). Synthesis, cytotoxicity, cell uptake and DNA cross-linking of 4,4′-dipyrazolylmethane-linked multinuclear platinum anti-cancer complexes. Anticancer Drug Des.

[CR24] Wisniewski Z, Surga WJ, Opozda EM (1994). Palladium(II) methylpyrazole complexes. Trans Met Chem.

[CR25] Zheng Y, Yi Y, Wang Y, Zhang W, Du M (2006). Preparation of chitosan–copper complexes and their antitumor activity. Bioorg Med Chem Lett.

